# Harnessing pro-inflammatory and immunopathologic immune responses in urinary tract infections for vaccine development: it’s all about a balance

**DOI:** 10.3389/fimmu.2026.1753331

**Published:** 2026-02-02

**Authors:** Sivakumar Periasamy, Joyce Lübbers, Susan King, Elise S. Hovingh, Leslie van der Fits, Germie P. J. M. van den Dobbelsteen

**Affiliations:** 1 Infectious Disease and Vaccine, Johnson and Johnson, Spring House, PA, United States; 2 Bacterial Vaccine Discovery & Early Development, Johnson and Johnson, Leiden, Netherlands

**Keywords:** AMR, immunopathology, mucosal vaccines, UPEC, urinary tract infection, uropathogenic *E. coli*, vaccines

## Abstract

Urinary tract infections (UTIs) cause a high economic burden with frequent medical visits, and in severe cases can lead to hospitalization due to complications like bacteremia or sepsis. UTIs are treated with antibiotics; however, this contributes to the emergence of antimicrobial resistant (AMR) bacterial strains because of misuse and overuse of antibiotics. *Uropathogenic E. coli* (UPEC) is the most common cause of UTIs and is commonly associated with antibiotic resistance. Several host defense mechanisms including the urothelial barrier, antimicrobial peptides, and complement protect the urinary tract from infection. If UPEC is encountered, a pro-inflammatory immune response starts to combat the infection, with antimicrobial peptides and protein as a first line of defense followed by the activation of the innate and adaptive immune responses. These innate and adaptive immune responses are sometimes inadequate during established UTI, and recurrence of UTI is common. In addition, an overactivation of the immune response to UPEC causes immunopathologic damage to tissues and cells. Anti-*E. coli* vaccines have been proposed as an ideal approach both to improve the immune response to infection and to limit the emergence and spread of AMR strains. Currently, a few UTI vaccines have been licensed in a couple of countries but are not broadly approved and novel vaccines are being explored. In this review, we focus on the pro-inflammatory response to UPEC infections and the immunopathologic effects of an overactive pro-inflammatory response during UTIs in humans. We highlight the components of the immune response during UTI that can be utilized for the development of a preventative UPEC vaccine.

## 1 Introduction

Urinary tract infections (UTIs) have a high prevalence and are one of the most common bacterial infections worldwide. In current clinical practice, antibiotics are often used to manage UTIs, but this also results in the emergence of antimicrobial resistant (AMR) bacterial strains worsened by misuse and overuse of antibiotics. AMR is common in many bacterial infections like bloodstream, respiratory, and intra-abdominal infections, and can have fatal consequences. An extensive study investigating estimated deaths and disability-adjusted life years attributable to and associated with bacterial AMR in 2019 estimated a total of 58.1 deaths per one hundred thousand people in the European region, where 5.2 of those were a consequence of UTIs (1). Next to increased deaths associated with AMR, UTIs also account for a high economic burden due to frequent medical visits or, in severe cases, hospitalization. The difference in UTI related costs in the USA between uncomplicated UTIs with antibiotic susceptible bacterial strains ($991 in 2020) and antibiotic non-susceptible urinary isolates ($1147 in 2020), treated in an outpatient clinic over a duration of 6 months, was approximately 15 percent (2). Bigger differences were found in a UK study where hospitalization for progression of the uncomplicated UTI to complicated UTI was included in the calculations (3). If a patient has recurrent UTIs (rUTI), the costs rise with multiple visits, workup costs, and treatment costs. In addition, antibiotic resistance can increase these costs significantly by requiring specific intravenous antibiotics to treat UTIs (4). A recent national database study in the USA showed that there were over 2.3 million UTI cases per year with 700,000 complicated UTI cases that together resulted in annual 30-day total costs of more than $7.6 billion (5). These costs to treat UTIs are expected to increase in the coming years due to the rapid emergence of AMR strains that do not respond to the first line of antibiotic treatment.

UTIs are mainly caused by *Uropathogenic Escherichia coli* (UPEC), a pathotype within the pathogroup of extra-intestinal pathogenic *E. coli* (ExPEC) (6). Most UPEC strains originate from the gut and ascend through the urinary tract to the bladder to cause UTIs. The clinical spectrum of UTIs includes acute, chronic, and recurrent infections with urological manifestations such as cystitis (bladder infection), prostatitis (prostate infection), pyelonephritis (kidney infection). UTIs can also manifest as asymptomatic bacteriuria (6). UTI can lead to severe invasive *E. coli* disease (IED) with conditions like bacteremia (bloodstream infection) and sepsis, which have a potentially high mortality rate. The risk factors for developing UTIs are dependent on the population, co-morbidities present in the population, and can be behavioral, anatomical, or genetic in nature. Looking at biological sex, UTIs occur within all sexes but are more frequent in women due to a shorter urethra. UTI risk factors for women are linked with frequency of sexual intercourse, perineal hygiene (washing, material of underwear, menstrual hygiene), age and pregnancy (7, 8). More general risk factors are anatomical abnormalities, transplantations (due to immunosuppressive medication), urinary catheterization, and polymorphisms in genes involved in the immune response against bacteria (7, 8).

When colonization of the bladder with UPEC happens, an immune response starts with the recognition of the pathogen associated molecular patterns (PAMPs) on *E. coli* by pattern recognition receptors (PRRs) such as Toll like receptors and the inflammasome components that are expressed by innate immune cells. This activates an immune response, which in most cases controls the infection and leads to development of a memory response, referred to as protective immunity (9). On the other hand, sometimes an infection triggers overactivation of the immune system, leading to damage of tissues and cells referred to as immunopathologic or pathologic response (10). UPECs are shown to trigger several immune defense mechanisms in the bladder and kidneys during UTI (11), but treatment with antibiotics is often necessary to clear the infection. This indicates that the natural immunity is probably weak and transient or is skewed towards an immunopathologic response following UPEC infection in humans. Notably, as most of our understanding of immune responses during UTI is derived from experimental animal models, a complete understanding of the human immune response to UTI is lacking. In addition, immune correlates of protection are not well-defined in UTI making research into treatment and prevention of UTIs complicated.

Research into the treatment of UTIs is mainly focused on alternatives to antibiotics including small molecules targeting bacterial adhesins (e.g., mannosides, galactocides), ureases (e.g., acetohydroxamic acid), probiotics, nutraceuticals (e.g. cranberry juice, herbal medicine) and immunomodulatory approaches. Many of these treatments have been evaluated for their protective properties against UTIs in animal models, but results vary heavily between treatments, and not all have progressed to clinical trials (12–16). Another approach to combating UTIs is an active immunization regimen against UPEC, which can prevent UTI or rUTI by boosting the immune system of populations susceptible to UPEC. Currently, a few UTI vaccines have been licensed in a couple of countries (17, 18) but are not broadly approved and novel vaccine are being explored. The biggest challenge for UTI vaccines is to get an adequate immune response in the bladder to prevent UPEC infections. Therefore, a better understanding of the human immune response during UTI could shed light on the type of immunity that is required for protection against UTI after vaccination. This will inform the design of antigens and/or adjuvants that promote the desired immune response. In this in-depth review, we provide insights on current understanding of pro-inflammatory/protective response and immunopathologic/pathologic response elicited during UTIs in humans. We then discuss how to harness these insights to formulate working guidance for the development of a vaccine aimed at prevention of UTIs caused by UPEC.

## 2 UPEC strategies for infection, colonization, and immunomodulation

To develop effective vaccines for preventing or treating UTIs, it is essential to understand how UPEC colonizes and infects the urinary tract. When UPEC strains are present in the gut, for example, they enter the urethra and move upward into the bladder where they colonize the urinary tract. Variety of virulence factors promote bacterial adherence, colonization, persistence, and modulation of the host immune response. Additionally, UPEC evades host defenses by forming biofilms or intracellular bacterial communities (IBCs), which act as protective niches and reservoirs for recurrent infections (19).

### 2.1 Virulence factors

During the infection process, UPEC strains employ several virulence factors including fimbrial adhesins, flagella, LPS, K capsule, toxins, and iron-acquisition systems. These UPEC virulence factors can be categorized into surface virulence factors, and secreted virulence factors (20). The surface virulence factors allow for motility and adhesion to target cells and defend from attack by immune system components. Secreted virulence factors exert their effects on host cells to enhance access to target cells, nutrients, disarm or cause cell death of immune cells, and block soluble complement components.

#### 2.1.1 Bacterial adherence, motility, and colonization of urinary tract epithelium

**Figure 1:**
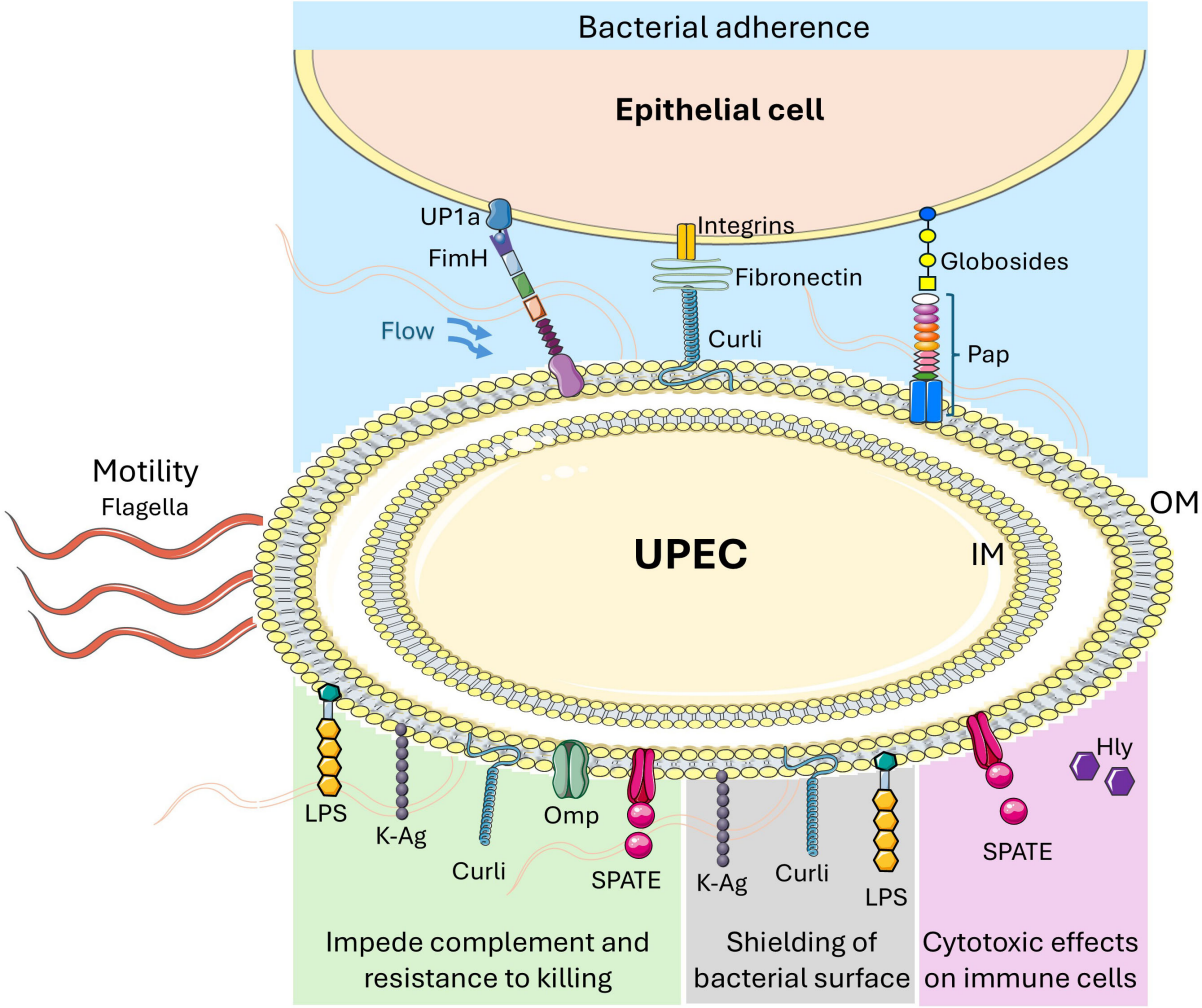
*Uropathogenic E. coli* (UPEC) adherence, motility and immunomodulation strategies. UPEC uses different adhesins for adherence to bladder epithelial cells (light blue box), for example, type 1 fimbriae FimH that binds to uroplakin UP1a in a catch bond way to successfully colonize and move upward in the urinary tract under urine flow or urine voidance situation, Curli fibers that bind among others to fibronectin, and P fimbriae (Pap) that bind to Globosides. Flagella on UPEC is mainly used for upward bacterial motility and movement within the urinary tract. Although bacterial attachment to urinary tract epithelial cells activate different arms of host immune responses, a variety of immunomodulatory components and mechanisms that are deployed by UPEC impede them. For example, bacterial capsule, LPS, curli and other cell membrane components inhibit complement activation and confer resistance to host immune cell killing (light green box), to restrict host immune cell access to the bacterial surface (grey box), and/or to have a cytotoxic effect on host immune cells (light pink box). LPS = lipopolysaccharide, K-Ag = K capsule antigen, Omp = Outer membrane proteins, SPATE = soluble serine protease autotransporters, Hly = α-hemolysin, IM= inner membrane, OM= outer membrane. Figure created by adapting and using images from Servier Medical Art (https://smart.servier.com/), licensed under CC BY 4.0 (https://creativecommons.org/licenses/by/4.0/).

UPEC have multiple adhesins like the type 1 fimbriae (fim), P fimbriae (pap), F1C fimbriae (foc), S fimbriae (sfa) Dr fimbriae (dra) and curli fibers (csg) that can contribute to successful colonization of epithelial cells of the urinary tract. FimH, an adhesion subunit of type 1 fimbriae, seems to be the most prominent of the adhesins in UPEC strains and is located at the tip of the fimbriae **Figure 1** (21). FimH makes direct contact with terminal mannosyl moieties of uroplakin UP1a present on bladder mucosa through a ‘catch-bond’ interaction, which simulates molecular hooks. In static urine conditions three molecules of FimH can bind UP1a with a low to moderate affinity, but these molecular hooks hold firm with a monovalent high affinity bond when stretched by tensile force, such as urine flow (22, 23). Type 1 fimbriae, like all glycoproteins that contain terminal mannosidases, can bind extracellular matrix and mediate bacterial uptake into cells via β1 and α3 integrins. In addition, UPEC flagellar activity helps upward bacterial motility which could account for the successful colonization of the urinary tract by UPEC when compared to non-flagellated bacteria **Figure 1** (23–25).

#### 2.1.2 Host immune modulation

*E. coli* virulence factors serve to evade host defense mechanisms but may also be the target of immune responses, and as such play a critical role in UPEC immunity and pathogenesis. The strategies to evade host defense mechanisms depicted in **Figure 1** include: a large number of surface and secreted virulence factors that impede the action of the complement system, including LPS, K capsule, outer membrane proteins Omp, and soluble serine protease autotransporters (SPATEs) (26–29); virulence factors that protect the bacterial cell surface from complement binding or activation such as LPS, colanic acid, and Curli (30–32) and surface and secreted virulence factors that provide resistance to cell-mediated killing such as LPS, K capsule, Lpp, and SPATEs (28, 33, 34). The multiplicity and redundancy of these virulence factors hint at the multitude of immune mechanisms that are needed to keep *E. coli* in check. Virulent UPEC strains also employ several mechanisms that suppress pro-inflammatory cytokines and inflammatory processes of the host (35, 36). For instance, attachment of the secreted UPEC toxins like α-hemolysin or SPATEs to host cell membranes triggers proteolysis of host proteins and cell death in epithelial and immune cells that results in suppression of the inflammatory response (37). On the other hand, UPEC can take advantage of host immune responses. For example, pro-inflammatory cytokines have been shown to enhance the growth of UPEC strain CFT073 in broth culture, and activate genes associated with iron acquisition or other virulence factors that may potentiate bacterial survival during infection (38).

### 2.2 Extracellular bacterial biofilm

Many extracellular bacteria form biofilms, in which bacterial cells assemble in a tight community embedded in an extracellular matrix. The biofilm serves multiple purposes: metabolic adaptation, allowance of a dormancy state of bacteria, a barrier from antibacterial host factors/drugs, and a source for acute infection or re-infection. Biofilm formed by UPEC strains in a urinary catheter or in the urinary tract is the main barrier for host immune cells and antimicrobial drugs to counter bacterial replication (39). *In vitro* studies showed that highly virulent UPEC clinical isolates formed a thin biofilm, whose biomass was unaffected by neutrophils. In contrast, strains with a limited set of virulence factors formed a thick biofilm, but neutrophils could reduce their biomass (40). Biofilm formation by UPEC strains is linked to recurrent UTIs in patients and is a complicating factor for the successful treatment with antimicrobial drugs (41, 42).

### 2.3 Intracellular bacterial communities

Although UPECs are mainly extracellular, some strains could form intracellular bacterial communities (IBC) in bladder epithelial cytoplasm. Bacterial IBC not only offers a protective niche from antibacterial peptides, immune cells or antibiotics, but could also serve as a source for recurrent infection (19, 43, 44). Human bladder on chip and bladder organoid models showed IBC formation and delayed bacterial clearance by antibiotics within the IBC (45, 46). Exfoliated IBCs were noted in urine sediments of some UTI patients (47). Once IBCs are established, they may predispose the individuals to rUTI, suggesting that UPEC is adaptable for intracellular survival within the epithelial cells.

## 3 Urothelial and bactericidal host defenses

UPEC uses multiple virulence mechanisms and resistance factors to infect and colonize the urinary tract epithelium. Several host defense mechanisms including the urothelial barrier, antimicrobial peptides, and complement protect the urinary tract from infection.

### 3.1 Urothelial barrier

The urinary tract is exposed to environmental microbes, as are the gastrointestinal and respiratory systems. Unlike the gastrointestinal and respiratory tracts, the inner wall of the urinary tract is covered by an unusually thick mucosal layer called the urothelium. Urothelium is composed of urothelial cells that are arranged in multiple layers including basal, intermediate, and superficial layers. The superficial umbrella cells fuse closely, forming an impermeable epithelial surface, which is impervious to fluids, inert particles, and infectious agents. In addition, umbrella cells express urothelium-restricted glycoproteins (uroplakins) forming superficial plaques, and mucopolysaccharide-rich glycosaminoglycan layers on the apical surface (as reviewed in (48)). FimH binding to urothelial uroplakins facilitates attachment, colonization, and early invasion (within 2 hours) of UPEC as shown in mice and *in vitro* human bladder cell models. As an initial response to bacterial attachment, bladder epithelial cells shed from the mucosal surface through cell death and this exfoliation can start within 2 hours and continues for at least 48 hours post infection (19, 49–51). Simultaneously, bacteria can re-emerge from the dead cells and re-infect neighboring cells, leading to a sustained infection as shown in mice (19). Exfoliation is partly dependent on the bacterial toxin α-hemolysin to stimulate caspase dependent cell death and on FimH-uroplakin binding to activate this same intrinsic apoptotic pathway in urothelial cells (51, 52). Not all cells with attached bacteria will exfoliate. Bacterial attachment can also trigger epithelial regeneration. For example, FimH activates positive regulators of cell proliferation and tight junction components, and down-regulates inhibitors of epithelial regeneration. This helps to rapid regeneration of lost epithelial cells (53). Nevertheless, neither the urothelial barrier nor exfoliation of infected urothelial cells are sufficient to prevent bacterial colonization during UTI.

### 3.2 Antimicrobial peptides and glycoproteins

Antimicrobial peptides (AMPs) are critical components of the innate immune system. In the urinary tract, AMPs are secreted by uroepithelial cells to maintain sterility by limiting bacterial attachment, depleting nutrients required for bacterial growth, and attracting leukocytes (54). In healthy individuals, cathelicidins, ribonuclease 7 (RNase 7), α-defensin and β-defensin 1 are constitutively expressed by the uroepithelium. Most of these AMPs are expressed in higher concentrations during UTI infections (54–56). Other AMPs that are expressed during UTI infections are lipocalin-2 (NGAL), lactoferrin, SA100A9, REG3γ, and pentraxin-3 (54). The level of β-defensin in urine was found to be highly correlative with the risk of UTI in females (57). Since the efficacy of AMPs is concentration-dependent, their impact on controlling UTIs in the presence of urine flow is uncertain.

Next to AMPs, urinary tract specific glycoprotein - the Tamm-Horsfall protein (Uromodulin) - is highly abundant in urine. The Tamm-Horsfall protein, produced by the kidney, plays an important role in regulation of homeostatic physiology in the kidneys and protection against UTI (58). With a genome wide association study, it was found that certain single nucleotide variations in the *UMOD* gene that encode the Tamm-Horsfall protein are associated with a higher concentration of Tamm-Horsfall protein in serum and urine, and a reduced risk of UTIs (59).

### 3.3 Complement

Complement components are ubiquitous in blood plasma and tissues. Activation of complement pathways controls bacterial replication by directly attacking the bacterial cell wall and by enhancing opsonophagocytosis, i.e., uptake and killing of antibody coated bacteria by phagocytic cells such as neutrophils and macrophages. However, *E. coli* employs several mechanisms to circumvent complement-mediated killing, including the surface expression of O and K antigens to shield complement binding sites, production of proteases to degrade complement proteins, and the production of bacterial factors that bind to Antibodies to prevent activation of complement pathways (60). Individuals with complement component deficiencies are less susceptible to disease caused by *E. coli* than by other capsulated bacteria such as *Pneumococci* spp. and *Heamophilus* spp (61). Since UTIs are less common in many primary immunodeficiency disorders (PID), as shown in **Table 1**, it is suggested that complement mediated direct killing is one of several immune mechanisms that are deployed in concert to control *E. coli* and counteract infection.

**Table 1:** Summary of primary immunodeficiency disorders (PID) and susceptibility to UTI in humans.

PID	Defect and phenotype (human)	Susceptibility to infection	References
Severe Combined Immunodeficiencies (cellular and humoral immune deficiencies)			
SCID	Defects in T, B and NK cells. Phenotypes: 1) T^-^B^+^NK^+^ 2) T^-^B^+^NK^-^ 3) T^-^B^-^NK^+^ 4) T^-^B^-^NK^-^	Susceptible to respiratory tract and gut infections early in life. Gram-negative bacterial sepsis is common, but occurrence of UTI is less common	(87, 88)
IL-7Receptor α deficiency	Defects in *IL-7RA* gene, Phenotype: T^-^B^+^NK^+^	Recurrent viral infections, but no UTIs reported	(218)
LAT (Linker for activation of T cells) deficiency	Defects in *LAT* gene Phenotype: T^-^B^+^NK^+^	Autoimmune manifestation and immune dysregulation with severe infections leading to early death of children. No UTIs reported	(219)
Nude SCID	Defects in *FOXN1* gene. Phenotype: T^low^B^+^NK^+^ with abnormal thymus	Eczema, severe erythroderma, severe diarrhea, and alopecia. No UTI reported	(220)
RAG deficiency	Defects in *RAG1* and *RAG2* genes Phenotypes: 1) T^-^B^-^NK^+^ 2) T^autologus^B^-^NK^+^ (Omenn syndrome)	Life threatening viral, fungal, parasitic, and bacterial infections from birth. No UTIs reported.	(221)
MHC I deficiency	Defects in *TAP1* and *TAP2* genes Phenotype: normal T^+^B^+^NK^+^	Bacterial respiratory tract and skin infections. No UTIs reported	(222)
MHC II deficiency	Defects in *CIITA, RFX5, RFXAP* and *RFXANK* genes Phenotype: CD4T^low^CD8T^+^B^+^NK^+^	Recurrent severe infections (virus, bacterial, fungal), diarrhea, liver disease, and autoimmune cytopenia. No UTIs reported	(222)
Ataxia telangiectasia (A-T)	Defects in *A-TM* gene Phenotype: Low Ig and T cells	Children with A-T had urinary bladder dysfunction, but UTI is not reported	(223, 224)
Wiskott-Aldrich syndrome (WAS)	Defects in *WASP* gene Phenotype: High IgM levels, neutropenia, thrombocytopenia, T and B cell function impaired	Recurrent infections are common. Lack of infections of the urinary system.	(225, 226)
Primary Antibody deficiencies			
CD40L deficiency/Hyper IgM	Defect in *CD40L* gene Phenotype: Hyper IgM, Low IgG	Severe and opportunistic infections including UTI	(227)
X-linked agammaglobulinemia (XLA)/Bruton disease	Defect in *BTK* gene Phenotype: B^-^ and agammaglobulinemia	Mostly susceptible to respiratory tract infections, skin, and ear infections. Some UTIs are reported	(228)
Common variable immunodeficiency (CVID)	Defects in various genes responsible for B cell function Phenotype: low IgG, IgA and IgM	Mostly susceptible to respiratory tract and gastrointestinal infections. Some UTIs are reported	(229–231)
Selective IgG deficiency	Defect unknown Phenotype: Low or absent IgG and its subclass	Recurrent or severe lung infections. No UTIs reported	(89)
Selective IgA deficiency	Defect unknown Phenotype: Very low to absent IgA with other isotypes normal	Mild infections in the respiratory tract, gut and UTIs reported	(89)
Primary Cellular deficiencies			
DiGeorge syndrome	Defect in chromosome 22q11.2 Phenotype: congenital anomalies of multiple organ systems in children	Children are presented with high incidence of anomalies in kidney and bladder, including hydronephrosis, hydroureter and bladder reflux, but low incidence of UTI	(232)
Kostmann’s syndrome	Defect unknown Phenotype: Severe neutropenia	Life-threatening infections by *E. coli* and *S. aureus*	(233)
Elastase deficiency (SCN1)	Defect in *ELANE* gene Phenotype: Neutropenia	Recurrent bacterial infections (e.g. E. *coli* and S. *aureus*) causing e.g. pyelonephritis	(84)
CXCR2 deficiency	Defect in *CXCR2* gene Phenotype: neutropenia	Some bacterial infections	(234)
Leukocyte adhesion deficiency (LAD)	Defects in β2 intergrin (LAD-1 type), SLeX (LAD-2 type) and Kindlin 3 (LAD-3 type) Phenotype: leukocytosis with defect in neutrophil mobility	Recurrent bacterial infections including UTIs	(235)
Chediak-Higashi syndrome	Defect in *LYST* gene (probably) Phenotype: hemophagocytic lymphohistiocytosis	Recurrent infections including UTI	(236)

PID, primary immunodeficiency disorders; SCID, severe combined immunodeficiency; UTI, urinary tract infection.

## 4 Protective innate immune responses

Host recognition of pathogen associated molecular patterns (PAMPs) on *E. coli* by pattern recognition receptors (PRRs) such as Toll-like receptors and the inflammasome leads to the release of inflammatory chemokines and cytokines. These cytokines promote the rapid migration and activation of innate immune cells including neutrophils, macrophages, and NK cells. These cells act to contain the UTI and potentiate the immune response by recruiting adaptive B and T cells to the site of infection.

### 4.1 Toll-like receptors

Several virulence factors of UPEC (LPS, fimbriae subunits and flagellin) act as PAMPs and engage membrane-bound Toll-like receptors (TLR) on innate immune cells and urothelium. Engagement of TLRs leads to cell activation and production of AMPs, chemokines, and cytokines. The recognition of LPS by TLR4 and subsequent activation of the TLR4 pathway differs in urothelium and monocytes. Urothelium lacks co-receptors CD14 and MD2 for LPS-TLR4 signaling and therefore produces less chemokines and cytokines than monocytes/macrophages (62). In addition, inherent repeated exposure to LPS can lead to a hyporesponsive state, which results in a sub-optimal immune response (63). Women infected with *E. coli* 83972 with enhanced adhesion through genetically modified papGX fragment or Fim gene clusters showed enhanced activation of TLR2, 4, 5, 7 and 8 compared with the wild type *E. coli* 83972 infection suggesting that recognition of P fimbria enhances the immune reaction (64). A reduced TLR4 expression, but not its adaptor proteins (TRIF, TRAM or Myd88), was found to be associated with higher susceptibility to asymptomatic bacteriuria (65). Also, polymorphisms in the *TLR4* gene (*A299G* and *A896G)* were associated with higher susceptibility to UTI in children and women (66–70). Next to TLR4, TLR5 is abundantly expressed in the bladder and important for the recognition of *E. coli* flagellin and *Tlr5* knockout mice were more susceptible to UTI infections (71). Women with a polymorphism in *TLR5* (*C1174T*) that abolishes TLR5 function have an increased risk of rUTI (68). In addition, TLR2 expressed on tubular cells was shown to be activated by UPEC and induce an immune response. Polymorphisms in this gene (*G2258A* and *A753G)* and associated reduction in TLR2 signaling were associated with an increased risk for asymptomatic bacteriuria in women and recurrent UTI in children (68, 70). Also, other TLR polymorphisms like *TLR1 G1805T* and *TLR4 A896G* have been implicated to susceptibility to UTI (66–70).

### 4.2 Inflammasome

UPEC virulence factors such as LPS, Fim and Pap can activate PAMPs which leads to the assembly and activation of the inflammasome, which is a multiprotein intracellular pattern recognition receptor. The inflammasome is an integral part of the innate immune system which plays a key role in processing pro-form (pro-IL-1β and pro-IL-18) of cytokines, pyroptosis and immunosurveillance (72, 73). UPEC induction of the release of cytokines IL-1β and IL-18 can activate the immune system. Pyroptosis, a special form of cell death, leads to release of cytokines and cell components that compound or sustain the inflammatory response. Pyroptosis can also lead to urothelial cell death and thereby diminishes intracellular bacterial community locations (74). Activation of inflammasome components in immune cells and urothelial cells, including NLRC4, NLRP3, NAIP/NLRP1, ASC, and Caspase-1, but not AIM2 and Caspase-4, was reported in UPEC-infected UTI patients (75). NLRC4 is unique in that it recognizes bacterial flagellin, while NLRP3 recognizes a wide range of PAMPs.

### 4.3 Neutrophils

Neutrophils are recruited as the first-line innate defense. Following UPEC infection, neutrophils appear in the bladder as early as 4–6 hours post infection and utilize several antibacterial machineries to restrict bacterial replication, including phagocytosis, degranulation, and the formation of neutrophil extracellular traps (NET) (76, 77). Even before the discovery of TLR4 as the receptor for bacterial LPS, the Svanborg-Eden group demonstrated that UPEC LPS is a main component that induces recruitment of neutrophils that constitute the primary defense in animal models of UTIs (78, 79). Supporting this, neutrophils defective for phagocytosis and other bactericidal functions predispose patients to rUTI (80). NET formation by neutrophils was increased in children with UTI and can serve as a potential biomarker of infection (81). This NET formation during UTI is enhanced by Tamm-Horsfall glycoprotein that is abundant in urine (82). In elderly adults, weakened functions of neutrophils are linked to UTI (83). In addition, individuals with innate immune cell deficiency, specifically neutrophils, are more susceptible to UTI (84–86). However, people with combined immunodeficiency disorders (CID) or antibody deficiencies are less susceptible to UTI (87–89) (**Table 1**). A well-studied human genetic predisposition to UTI is acute pyelonephritis (APN), which includes a low expression of CXCR1 (receptor for neutrophil recruiting cytokine IL-8). Interestingly, CXCR1 is required for neutrophil recruitment to both bladder and kidneys, whereas APN-prone family members are highly susceptible to kidney infection compared with bladder infection (81, 90). Altogether, cumulative data emphasizes the protective role of neutrophils during UTI.

### 4.4 Macrophages and dendritic cells

Two main antigen presenting cells (APC), macrophages and dendritic cells (DCs), play a critical role in innate immunity and bridging innate responses to adaptive immunity. Tissue resident macrophages are the main players to initiate the early events of innate immunity, along with local epithelial cells (91, 92). Tissue resident macrophages are slowly replaced by monocyte-derived macrophages after infection with UPEC (93). After an initial wave of neutrophils that are recruited upon UPEC infection, monocyte-derived macrophages accumulate at the infection site between 24- and 72-hours of post-infection and extend the inflammatory process. However, as an innate sentinel, macrophages play multiple roles - including bacterial uptake, antigen processing and presentation, cytokine release and activation of adaptive immune cells – that are critical for controlling bacterial colonization/replication and immunopathogenesis. They are also implicated in curtailing the inflammatory response. Conventionally, macrophages can be categorized as classically activated (M1) and alternatively activated macrophages (M2) types. M1 macrophages are critical for the pro-inflammatory response, while the M2 phenotype is important for resolution of inflammation and an anti-inflammatory response (94). Both M1 and M2 macrophages are identified during UTI in the bladder (93). UPEC virulence factor TcpC skews the M1 vs M2 phenotype, specifically inhibition of M1 and promotion of M2 macrophages (95). DCs prime adaptive immune response to UPEC. However, high numbers of macrophages in the inflammatory milieu could impede the functional capacity of DCs during UTI (92).

### 4.5 Natural killer cells, innate lymphoid cells

There is limited information on the role of other innate immune cells such as natural killer (NK) cells and innate lymphoid cells (ILC) in human UTI. In a mouse UTI model, NK cells accumulate in the bladder on day 1 and 2 post-infections, attracted by stromal-cell derived factor 1 (SDF-1) from uroepithelial cells, but disappear on day 5. However, the number of NK cells accumulating in the bladder upon infection was lower than that of other innate cells (96–98). These NK cells cleared bacterial infection through TNFα secretion (96). In humans, it was observed that a single nucleotide polymorphism in FcγR3A (CD16a), the main Ab receptor on NK cells, was associated with susceptibility to UTI in rheumatoid arthritis patients treated with methotrexate or etanercept (99). Although ILCs accumulate in the bladder in very low numbers (~500 cells/bladder in mice), group 3 ILCs (lymphoid origin with IL-22 and IL-17 cytokine production) were shown to be protective during UPEC infection by early recruitment of neutrophils through IL-17A (97).

### 4.6 Trained immunity

Evidence of memory responses in innate immune cells and non-immune cells (e.g., epithelial cells) has accumulated and is termed as ‘trained immunity’. The mechanism behind this includes metabolic changes and epigenetic alterations in the promoter regions of inflammatory genes after activation of cells by PAMPs, that allow a faster and stronger recall response (100, 101). It was shown that the training of urothelial cells by primary UPEC infection could limit the recurrence of UTI (102, 103).

## 5 Protective adaptive immune responses

In addition to the innate immune response, the adaptive immune response mediated by B and T cells is crucial for prevention and containment of infection through specific antigen recognition and the generation of memory cells. Bacterial antigens are presented to B and T cells in draining lymph nodes, leading to local and systemic responses. Further, resident memory cells activated upon antigen recognition mediate long-term protection in the urinary tract. Antibodies produced by B cells inhibit UPEC adherence and survival through various mechanisms. T cell subsets secrete cytokines to enable homing and proliferation of effector cells during UPEC infection and are needed for the generation of high affinity antibodies by B cells.

### 5.1 Antibody response

Although several animal and human studies emphasize that innate immune cells (specifically neutrophils and macrophages) are sufficient for bacterial clearance during UTI (80, 91), the importance of the antibody response in human UTI was recognized decades ago (104, 105). Systemic as well as local presence of UPEC-specific antibodies and B cells in the bladder have been reported during UTIs in females (106). UPEC-specific antibodies cleared infection and favored resolution of cystitis in cynomolgus monkeys, in which IgM, IgG and IgA were found in serum, and IgA and IgG in urine (107). Antibodies inhibit UPEC adherence and colonization through several antibody-mediated mechanisms such as neutralization, opsonophagocytosis/antibody-dependent cellular phagocytosis, antibody-dependent cellular cytotoxicity and complement activation (described in section 3.3) (108). Antigen binding/neutralization is mediated through the Fab region, while cellular- and complement-mediated functions rely on binding to the Fc region of the antibody. Both T cell-dependent (antigen specific) and T cell-independent (not antigen specific) B cell responses are generated during UPEC infection. In *E. coli* infections, the bacterial O-antigen that covers the complete surface of the pathogen is a primary target for the host’s antibody response (105, 109) and typically activates T-cell independent B cells (106, 107). Studies have reported that the antibody response is different in the serum versus urinary tract during UPEC infection in humans (104). *E. coli*-specific antibodies are detected in the urine of UTI patients; however, patients with bladder infections induce less measurable pathogen-specific antibodies compared with those with kidney infections (110, 111). For example, IgG, IgA, and, in some cases, IgM antibody-coated bacteria were clearly identified in the urine of acute and recurrent pyelonephritic cases, but less in cystitis cases (112). This could be due to poor B cell homing, lower B cell diversity, or impaired antigen presentation in the bladder. The bladder lacks mucosa associated lymphoid cells, and it has a thick mucosal layer that could result in delayed or impaired B cell homing (113). In addition, a subset of DCs (tolerogenic) preferentially activates Th2 cells that are reported to inhibit antibody response during bladder infections in mice (114). On the other hand, local induction of Th1 cells in the bladder induced a protective antibody response (115). Another possibility is the impairment of DC antigen presentation through suppression by macrophages resulting in non-sterilizing adaptive immunity. Depletion of bladder-resident macrophages prior to infection in a mouse model improved DC function and the antibody responses (92). Although IgG, IgA, and IgM antibody responses during UTI are induced, they are not always sufficient to combat UTI.

Secretory IgA (sIgA) is abundantly present, highly stable, and resistant to host or microbial enzymes at mucosal surfaces. Uniquely, sIgA has oligosaccharide chains of the high mannose type which bind to the type 1 fimbria and S fimbria of *E. coli* and inhibit bacterial adhesion. sIgA engages in both Fab-dependent and Fab-independent binding of *E. coli* lectins (116, 117). Through this, sIgA is thought to play a critical role in preventing bacterial colonization and invasion at mucosal surfaces. Supporting this, the presence of higher levels of sIgA in the urine of UTI patients demonstrated a higher *in vitro* inhibition of UPEC adherence to urothelial cells (118). As sIgA lacks a C1q binding site, it is not effective in activating the classical complement pathway, but it could still activate the alternative and mannose-binding lectin pathways for complement activation (119).

### 5.2 T cell response

The CD8^+^ and CD4^+^ T cells mediate a protective response during UPEC infection by cytokine secretion and cell-cell interactions with B cells (19, 98, 120). Splenocytes from mice infected transurethrally with *E. coli* had increased levels of the activation marker CD69 on both CD8^+^ and CD4^+^ T cells. Adoptive transfer of these splenocytes or column enriched T cells resulted in reduced bacterial colonization of mice bladders in the recipients (120). The activation of CD4^+^ T cell subsets Th1, Th2, and Th17 ensures proper homing of effector cells and B cells to mucosal sites including the bladder, and Th1 and Th2 cells are needed for antibody class switching. While a Th1 response promotes UPEC clearance after an initial infection, Th2 cells inhibit bacterial clearance but promote urothelium repair (114). Th17 cells produce IL-17, a pro-inflammatory cytokine that promotes macrophage and neutrophil migration (121).

The Th1, Th2, and Th17 cellular responses develop during UTI. Where the CD4^+^ T_FH_ (T_follicular helper_) cells are essential for B cell maturation in secondary lymphoid organs and class switching of the B cells for high affinity antibodies (122). For the development of tissue-resident memory T (T_RM_) cells, antigen persistence during UTI was necessary. T_RM_ cells are antigen specific memory CD4^+^ T cells and CD8^+^ T cells that reside in tissues and provide a rapid response to subsequent infection at local sites (123). These polyfunctional cells hold a potential for broad effector functions at mucosal sites including IFNγ and IL-17 production and recruitment of other T and B cells (124). Further, it has been emphasized that a local mucosal memory T cell response, but not the systemic response, is critical for the protection against UTI, because T_RM_ cells present in the bladder and kidney elicited a protective role against UPEC (123, 125). Specific CD4^+^ T_RM_ cells with a Th17 signature (T_RM_17 cells) were observed in kidneys, but their localization in the bladder is not known (126). Some other T cell subsets like the γδT and mucosal-associated invariant T cells (innate like cells) were reported to play a role in UPEC infections. The γδT cells were shown to be important for resistance against UPEC and these cells are reported to produce IL-17 during UTI (121, 127). Whereas the number of mucosal-associated invariant T cells were not changed, their functional capacity was impaired in patients with rUTI (128). Overall, a broad variety of T cell responses from Th1 to Th17; T_RM_ and γδT occur during UPEC infections.

## 6 Inflammatory mediators in UTI

Immune responses at infection sites are orchestrated by a complex blend of soluble and cellular components. Pro- and anti-inflammatory mediators play a vital role in shaping this response: several pro-inflammatory mediators, including different cytokines and eicosanoids, are associated with antibacterial mechanisms to rapidly curb UTIs, while anti-inflammatory mediators, the cytokine IL-10, are needed to control the excessive inflammatory response.

### 6.1 Pro-inflammatory mediators

Among several pro-inflammatory mediators, IL-8 and G-CSF are considered important for neutrophil recruitment in mouse models of UTI (129, 130). However, data from mouse studies do not always translate to human data. The level of urine IL-8 is correlated with neutrophil counts in human UTI (131–133), whereas polymorphisms in CXCR1 or CXCR2 (specific receptors for IL-8) are not associated with the risk to develop UTI in women (134, 135). G-CSF is important for neutrophil recruitment at the early phase of infection, but not at the later phase of infection, as there is no correlation between levels of G-CSF in serum and blood leukocytes in the late stage of UTI in patients (136). However, urine G-CSF level in women with a UTI was higher than that of healthy controls (137).

Prostaglandins and leukotrienes are lipid compounds (eicosanoids) catalyzed by cyclooxygenase (COX) and lipoxygenase (LOX) pathways, respectively. Prostaglandins are implicated in the neutrophilic inflammatory process and their inhibition protected mice from chronic and recurrent UTI (138). Elevated levels of prostaglandin E2 were associated with UTI and kidney disease in humans (139, 140). However, the inhibition of COX-2 did not favor the host response as it interfered with several defense mechanisms against UPEC (141).

Other pro-inflammatory cytokines such as TNFα, IL-1β, IL-6, and IL-17 are highly produced during UTI with a multitude of roles in bacterial clearance (62, 142–144). Interestingly, these cytokines also impact the expression of virulence factors and can increase UPEC growth in an *in vitro* setting (38). In a genetic analysis of rheumatoid arthritis patients treated with methotrexate or etanercept (TNF inhibitor) (99), single nucleotide polymorphisms in TNFα and lymphotoxin A (TNFβ) are associated with UTI susceptibility. IL-1β is activated by either inflammasome-dependent or independent mechanisms following UPEC infection, thereby activating innate immune cells, T cells, and B cells. UPEC strain CFT073 induced NLRP3 inflammasome-dependent release of IL-1β from human neutrophils. In addition, UPEC virulence factors such as α-hemolysin, type-1 fimbriae, and p-fimbriae also activated inflammasome-independent mechanisms to release IL-1β from neutrophils (145). IL-6 is upregulated in human urothelium in response to UPEC, aiding in neutrophil recruitment and the differentiation of Th17 cells (62). IL-17 is a pro-inflammatory cytokine that promotes macrophage and neutrophil migration. IL-17 is secreted by a range of cell types, including Th17 cells, CD8^+^ T cells, and γδT cells. Importantly, *Il17*^-/-^ mice have demonstrated a defect in UPEC clearance and are more susceptible to cystitis (121). In these mice, the CFT073 strain was not as successful as the EC958 strain in causing chronic infection due to differences in flagellin types (146, 147). Notably, humans with IL-17RA deficiency were found to be susceptible to bacterial infections of the lower respiratory tract or skin (by *Staphylococcus aureus* or *Candida* spp.) but were not susceptible to UTIs (148, 149).

### 6.2 Anti-inflammatory mediators

IL-10 is an anti-inflammatory cytokine and is a master regulator of inflammatory processes. Although individuals with IL-10 and IL-10R deficiencies are highly susceptible to inflammatory bowel disease, owing to a failure to control excessive inflammation (150, 151), no susceptibility to UTI has been described. In IL-10R-deficient patients, IL-1β drives intestinal immunopathology (152). In patients with rUTI, IL-10 levels are significantly increased in urine (143, 153, 154). Coculture experiments of human bladder uroepithelial cells, monocytes and lymphocytes infected with UPEC showed that both uroepithelial cells and monocytes were responsible for the production of IL-10 (143, 153, 154). IL-10 is transiently activated during sepsis/endotoxemia, during which IL-10 has a regulatory effect to control the excessive activation of pro-inflammatory cytokines and inflammation (155, 156). This underscores the importance of IL-10 in limiting inflammation.

## 7 Immunopathology caused by UPEC during UTI

Despite vast evidence that both innate and adaptive immune arms play a role in UPEC immunity, the relatively common occurrence of UTIs shows that the urothelium and urinary tract are penetrable by *E. coli*. The recurrence of UTI in the same individuals further implies that natural immunity is ineffective. This could be due to immune escape by UPEC and an ineffective immune response or skewing of the host immune response to a pathologic response. Immunopathologic responses can aid in the manifestation and recurrence of UTIs (10). We provide a comprehensive description of known immunopathologic responses observed during UPEC infection.

### 7.1 Urothelial barrier pathology

Although urothelium is a tough barrier, microbial invasion occurs when its barrier integrity is compromised. UPEC mediates urothelial apoptosis through both the intrinsic and extrinsic apoptosis pathways by inducing Caspase 3 activity (51). In addition, UPEC use aerobic glycolysis through oxygen scavenging inside host cells, which alters the metabolic pathways and induces urothelial cell death (157).

### 7.2 Antimicrobial peptides and Tamm-Horsfall protein in immunopathology

AMPs can contribute to immunopathology in the bladder. For example, cathelicidin could promote bladder inflammation despite being shown to inhibit bacterial growth *in vitro*. Cathelicidin-related antimicrobial peptide (CRAMP)-deficient mice showed higher susceptibility to UPEC but had less inflammation following UPEC infection, suggesting a pathological role for CRAMP stimulating more inflammation and bladder tissue damage (158).

Tamm-Horsfall protein is important for the maintenance of homeostasis in the kidney and bladder but is also associated with kidney stones and an increased rate of catheter associated UTIs. Low levels of Tamm-Horsfall protein and citrate are correlated to kidney stone formation in adults (159, 160), while abundant Tamm-Horsfall protein increases the risk of catheter associated UTIs by enhancing *E. coli* binding to the catheter (161). Autoantibodies against the Tamm-Horsfall protein are normally formed during UTIs, and low levels of these autoantibodies are correlated with renal scarring in adults and children with a history of UTIs (162). Thus, a delicate balance of Tamm-Horsfall protein in urine is necessary to decrease the risk of UTIs and also minimize the risk of kidney stones.

### 7.3 Innate immune cells in immunopathology

Although neutrophils and macrophages are critical for early immunity, virulent UPEC strains can overwhelm neutrophil defense and establish an acute symptomatic or chronic UTI. Severe inflammation at an early stage of UTI causes bladder pathology and predisposes to chronic cystitis (129). Neutrophil granules are rich in enzymes and pre-formed proinflammatory cytokines that inadvertently cause tissue damage. TLR4 deficient mice, lacking neutrophil infiltration upon *E. coli* CFTO73 infection, showed no abscess formation on the kidneys whereas WT mice showed extensive abscess formation (163). During infection, neutrophils release NETs, and these NETs contain α-synuclein (αSyn). This αSyn has been shown to form aggregates in the peripheral nerves of the bladder and kidney and is a risk factor for multiple system atrophy, a rare progressive neurodegenerative disorder affecting the brain and nervous system (164). Proteome analysis of clinical UTI samples showed relatively high quantities of innate immune defense proteins, primarily produced by the activated neutrophils (165), indicating that the appearance of more neutrophils directly correlates with the severe form of clinical UTI (166). Depletion of neutrophils in a mouse model of acute pyelonephritis leads to an accumulation of inflammatory macrophages (M1) in the kidneys of these mice leading to renal scarring (167). A careful balance of neutrophil and macrophage infiltration to initiate inflammation vs immunopathogenesis is warranted to control UPEC infection.

### 7.3 Adaptive immune cells and anti-inflammatory mediators in immunopathology

T cells mediate a protective response during UPEC infection by cytokine secretion and cell-cell interactions with B cells (19, 98, 120). During the infection, the inhibition of T cells or activation of anti-inflammatory mediators like IDO and IL-10 contributes to the persistence of UPEC infections and subsequent bladder pathology. T cells can be inhibited by DCs and macrophages upon the interaction between CD14 on the cells and the UPEC FimH (168). In addition, rapid induction of IDO (indoleamine-2,3-deoxigenase) and immunoregulatory cytokines like IL-10 by regulatory T cells, APC or mast cells negatively impact T cell activation (143, 153, 169, 170). IDO converts tryptophan, which is essential for T cell metabolism, into intermediate metabolite kynurenine which is toxic to T cells (169). In elderly individuals with rUTI, a higher urine level of IL-10 is correlated with persistence of *E. coli* and repeated episodes of UTI with increasing bladder damage (153). Another study demonstrated that human bladder epithelial cells promote IL-10 production in synergy with monocytes (154). While it plays a role in moderation of tissue damage caused by excessive or immunopathologic inflammatory response, IL-10 could also result in ineffective generation of *E. coli* antigen-specific T cells.

Another anti-inflammatory agent is transforming growth factor β1 (TGF-β) that downregulates the pro-inflammatory cytokines and thereby immunopathology. In children with UTI, a high level of TGF-β in urine is correlated with normal renal scans, while low or non-detectable TGF-β in urine was correlated with more abnormalities (fibrotic changes) on the renal scans (171). In addition, children with TGF-β polymorphisms leading to reduction or loss of function are more likely to have renal parenchymal scarring following UTI (172).

### 7.4 Inflammatory mediators in immunopathology

Various kinds of cytokines and chemokines play a role in the immune response during a UPEC infection. Some cytokines and chemokines are also involved in the immunopathologic response seen during UPEC infections. One of these cytokines is IL-1β that can be released upon inflammasome activation by UPEC strains (173). In acute cystitis, IL-1β drives hyper-inflammatory disease and severe pathology in the bladder of mice. Consistently, *Il1β*^-/-^ mice were protected from UPEC infection and had no macroscopic evidence of acute cystitis in the bladder (174), suggesting that excessive IL-1β is causing bladder immunopathology. If these data hold true for humans, the higher expression of inflammasome components and their activation by cell stress signals could precipitate UTI immunopathology in humans.

Another cytokine pathway involved in immunopathology is the TNFα receptor pathway. Depletion of TNFα with an antibody in mice that resolved a primary UTI infection, made these mice more susceptible to reinfection compared to their non treated counterparts (175). In the same study, it was found that the TNFα depleted mice have increased IBC formation due to less exfoliation of infected bladder epithelium, suggesting that reduced TNFα increases the severity of cystitis. While elevated pro-inflammatory cytokines and downregulated immunoregulatory cytokines induce a strong immune response to combat UTI, it can also cause severe immunopathology during a UPEC infection.

## 8 Vaccine strategy to combat UTI infections

Antibiotics are the first line of treatment for UTIs. However, with emerging antibiotic resistance, other approaches are warranted. One of the approaches is to prevent UTIs with vaccination against UPEC, which cause 70-80% of UTIs. The question remains what makes a good UTI vaccine? Good vaccines are expected to enhance broader and stable immunity, specifically at mucosal sites through a combination of specific target antigens and multipurpose adjuvants administered via the most convenient route. The process of having all these factors combined in one formulation remains challenging. Development and progress made on vaccine development against ExPEC have been nicely described previously (176). Additional to ExPEC vaccines, multiple anti-*E. coli* UTI vaccines have been evaluated in human clinical trials targeting adults with a history of rUTI with combinations of different vaccine formulations, adjuvants and immunization routes, as described in **Table 2**. A meta-analysis of data from UTI vaccine studies suggests that vaccines have a moderate effect on reduction of rUTI with minimal to no side effects (177). Currently, a few UTI vaccines have been licensed by individual countries or are available through expanded research programs (e.g., StroVac^®^, Uromune and Uro-Vaxom^®^) but are not broadly approved and novel vaccine formulations are being explored (**Table 2**).

**Table 2:** Vaccines tested to prevent or as prophylaxis for UTI or with an UTI endpoint in human clinical trials.

Vaccine + clinical study phase completed	Trial design and population	Antigen + Adjuvant	Administration route + regimen	Outcome	References
ExPEC4V phase 2, ExPEC10V and ExPEC9V (J&J) Phase 3	ExPEC4V safety and immunogenicity double blind placebo-controlled phase 2 trial in adults ExPEC10V and ExPEC9V safety, reactogenicity and immunogenicity double blind placebo- controlled phase ½ trial in adults, >60 years, with a history of UTI	4V, 10V or 9V *E. coli* O-polysaccharides bioconjugated to a detoxified variant of Exotoxin A from *Pseudomonas aeruginosa* (EPA) vaccine No adjuvant	i.m. injection 1 dose (0.5ml)	ExPEC4V phase 2 study showed significantly fewer UTIs (not serotype related) Safe and immunogenic and functional antibodies present for 9 O-antigen serotypes. J&J stated that no efficacy to prevent invasive *E. coli* disease in phase 3 clinical trial in participants with a history of UTI was found. No UTI related outcomes were announced.	(184, 186, 237, 238)
FimCH (SEQ-400) (Seqoia) Phase 1	Open label dose escalation phase 1 trial in females, 21–64 years, with history of rUTI	FimCH Adjuvant: PHAD (MPLA analog)	i.m. injection days 1, 30, 90, and 180 (0.3 or 0.5ml)	Safe and immunogenic (150-fold increase in antibodies) in healthy women with and without rUTI. 75% Reduction of UTI was seen in 12 months follow-up of 4 participants.	(189, 190)
Uro-Vaxom^®^ (OM-89) (OM Pharma) Phase 2 (Licensed in over 30 countries including Germany, Switzerland and Brazil as prophylaxis)	Double blind placebo-controlled phase 2 trial in females, 18–65 years, with a history of rUTI	Lyophilized mix of 18 different strains of *E. coli* No adjuvant	Oral capsule 3 months 1 daily, and first 10 days of month 7, 8 and 9	Significantly reduced UTI incidence in the first 12 months in females with a history of rUTI	(191)
Uro-Vaxom (OM-89S) (OM-Pharma) Phase 1/2	Double blind placebo-controlled phase 3 in females, 18–80 years, with a history of rUTI	Lyophilized mix of 18 different strains of *E. coli* With different lytic procedure to OM-89 No adjuvant	Oral capsule 3 months 1 daily capsule (10 days vaccine, 20 days placebo) And repeat at month 7, 8 and 9	No preventive effect on UTI after 6 or 12 months in females with a history of rUTI	(203)
UroVac ^®^ (Solco) Phase 2	Double blind phase 2 trial in UTI susceptible females	Heat killed *E. coli* (6 strains), *P. mirabilis, P. morganii, E. faecalis* and *K. pneumoniae* No adjuvant	Vaginal capsule suppositories weeks 0,1, 2, 6, 10 and 14	Increased the time between re-infection and moderate effect on infection rates in UTI susceptible females	(165, 166)
StroVac ^®^ (Strathmann GmbH) Phase 4 (Licensed in Germany as prophylaxis)	Double blind phase 3 trial in adults, 18–80 years, with rUTI Phase 4 open label prophylaxis efficacy trial in females with rUTI	Heat killed *E. coli*, *P*. *mirabilis*, *P. morganii*, *E. faecalis* and *K. pneumoniae* (Similar to UroVac) Adjuvant: Aluminum phosphate	i.m. injection days 0, 14 and 28 (0.5ml) Booster after 1 year	Overall reduction of rUTI from 5 to 1 per year in 12 months follow up. No significant difference with placebo due to the antibacterial effect of placebo (Dextran) used.	(17, 192)
Uromune (MV140) (Immunotek) Phase 3 (Licensed or available with expanded access programs in 26 countries e.g. UK, Spain, Australia)	Double blind placebo-controlled phase 3 trial in females, 18–75 years, with history of rUTI	Whole cell lysate of *E. coli, Klebsiella* spp.*, Proteus* spp. And *Enterococcus* spp. No Adjuvant	Sublingual spray Daily for 3 months	Significant reduction of UTI occurrence for 12 months in 38-90% of rUTI patients	(18, 193, 194)

i.m., intramuscular; UTI, urinary tract infection.

### 8.1 Immune enhancement by vaccination

**Figure 2:**
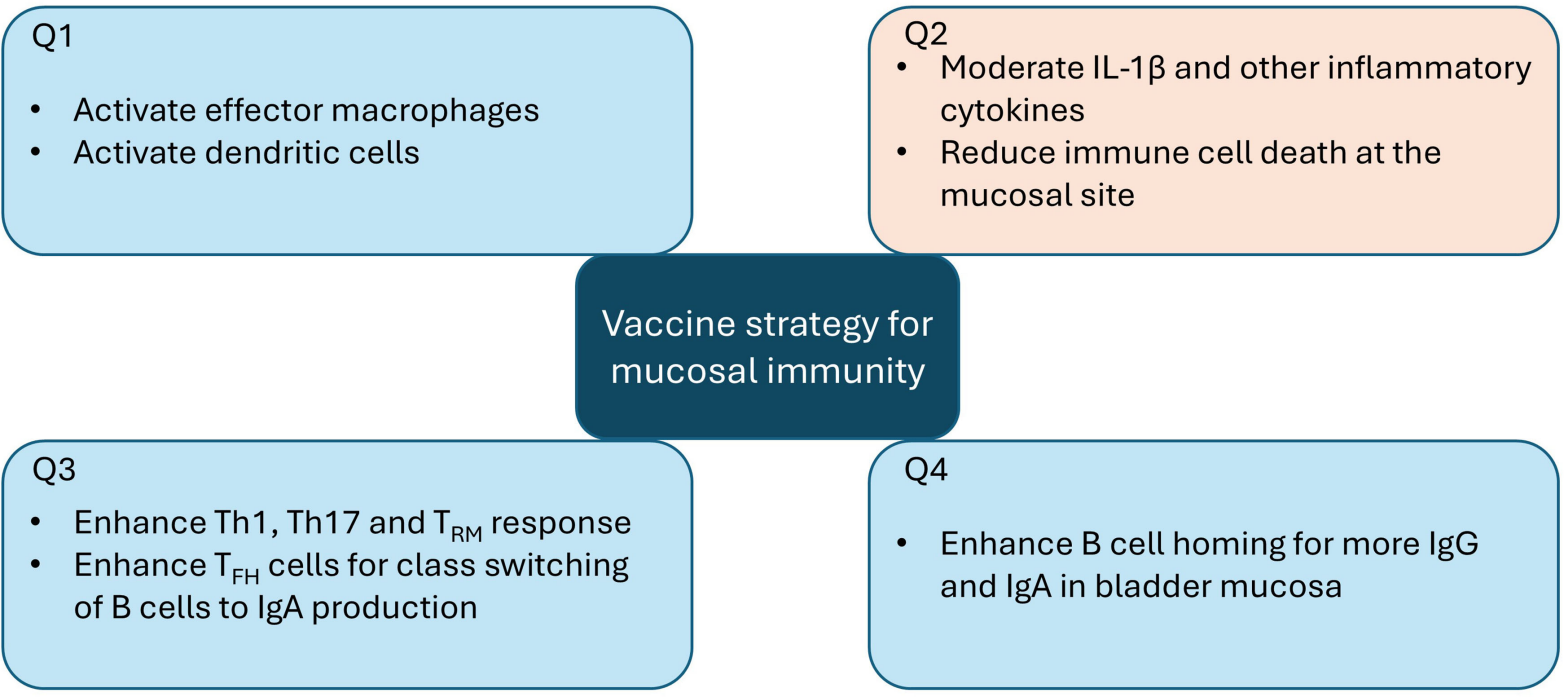
Vaccine strategy to enhance mucosal immunity against UTI and decrease immunopathology. The vaccine strategy must focus on increasing protective immunity (depicted in light blue boxes) and at the same time reducing the pathologic responses (depicted in the orange box).

As described in previous sections, UPEC has a complex relationship with the immune system, and no clear correlates of protection associated with either prevention of infection, symptom reduction, or limiting pathology have been identified. A vaccine that orchestrates a protective immune response to control infection while limiting excessive immunopathology is warranted (**Figure 2**). Proper activation of macrophages and DCs is necessary to take up antigens and prime CD4^+^ T helper, CD8^+^ cytotoxic T cells as well as B cells. Activation of effector macrophages and DCs could balance the inflammatory versus immunosuppressive effect (**Figure 2 Q1**) and limit the level of IL-1β and other pro-inflammatory cytokines that are implicated in an immunopathologic response (**Figure 2 Q2**). In addition, this process will also limit the rate of immune cell death, which will help in sustaining effector cells at the mucosal sites (**Figure 2 Q2**). In the adaptive immune arm, Th1 cells are shown to be effective in the bladder to control UPEC infections (114, 178) (**Figure 2 Q3**). Th17 cells and T_RM_ cells produce IL-17, a pro-inflammatory cytokine that promotes macrophage and neutrophil migration (121, 124) (**Figure 2 Q3**). By increasing antigen load through vaccination, the development of T_RM_ cells could be boosted. The CD4^+^ T helper cells ensure proper homing of effector cells and B cells to mucosal sites including the bladder. An optimal homing of B cells, either directly by vaccine or vaccine-adjuvant combination or indirectly by a swift cytokine response, and Th1/T_FH_/T_RM_ mechanisms could enhance a high titer of antibodies (both IgG and IgA) at the mucosal sites (**Figure 2 Q4**). Both IgG and IgA were found in urine and contributed to clearance of UPEC infection during cystitis in cynomolgus monkeys (107).

#### 8.1.1 Antibody response upon vaccination

**Figure 3:**
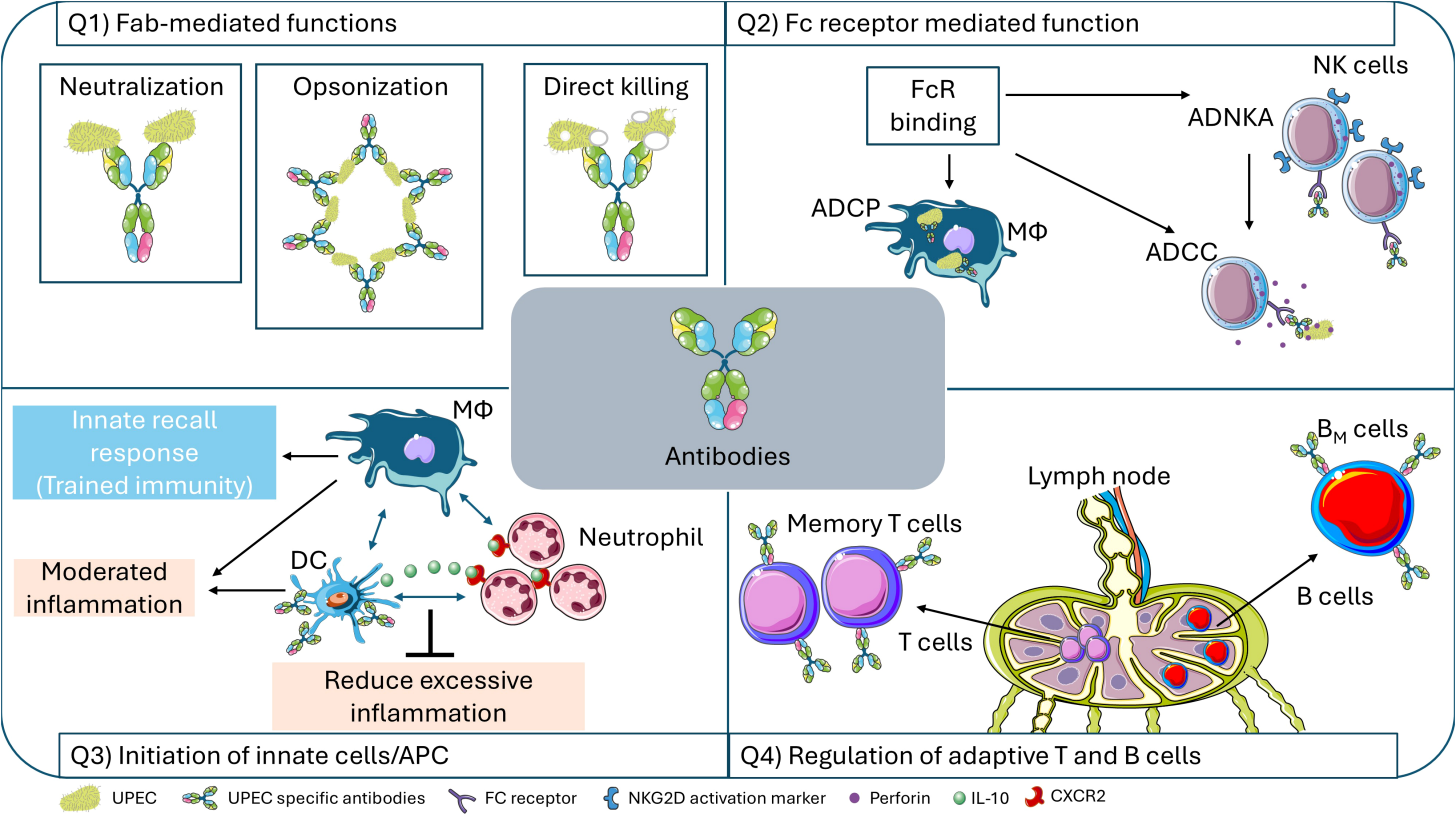
Multi-facets of antibody effector functions. Antibodies generated (after vaccination) or that are boosted rapidly upon infection can exert multiple roles. (Q1) Fab-mediated neutralization, opsonization and direct killing of pathogens. (Q2) Fc receptor mediated functions antibody-dependent cellular phagocytosis (ADCP), antibody-dependent NK cell activation (ADNKA) or antibody-dependent cellular cytotoxicity (ADCC). (Q3) Engagement of antibodies with innate cells and antigen presenting cells (DCs and macrophages) modulates their activation status to induce a more tolerogenic state. Tolerogenic DCs release IL-10 to inhibit neutrophil recruitment through the CXCR2 receptor. (Q4) Antigens processed by APC in the regional lymph nodes activate specific T and B cells to induce memory T cells and memory B cell (B_M_) expansion. Simultaneously, antibodies binding to antigens form Ab-antigen complexes that are taken up by DCs and activate follicular T cells (T_FH_) which further activate memory B cells. The protective immunity response is depicted in light blue box, and the pathological responses are depicted in orange boxes. Figure created by adapting and using images from Servier Medical Art (https://smart.servier.com/), licensed under CC BY 4.0 (https://creativecommons.org/licenses/by/4.0/).

Importantly, vaccine-induced antibodies could play multifaceted beneficial roles during infection. An optimal antibody response elicited by vaccination could clear UPEC by Fab-mediated neutralization, direct killing and opsonization or by Fc-mediated killing. Ab-mediated neutralization incapacitates pathogens by stripping off or neutralizing virulence factors or preventing cell tethering (**Figure 3 Q1**). Antibodies elicit Fc-mediated innate effector functions such as antibody-dependent cellular phagocytosis (ADCP) (179), antibody-dependent NK cell activation (ADNKA) followed by antibody-dependent cellular cytotoxicity (ADCC) (180), and activation of the classical complement pathway (**Figure 3 Q2**). Antibodies could also directly modulate the functions of innate antigen presenting cells (DCs and macrophages) to balance the inflammatory process and to counterbalance immunopathology (**Figure 3 Q3**). Importantly, antibodies form complexes with antigens, which are carried by DCs to regional lymph nodes to present antigens to follicular T helper cells (T_FH_). T_FH_ cells facilitate generation of memory B cells expressing high affinity antibodies (181) (**Figure 3 Q4**). Thus, immune enhancement by vaccination is directed at multiple parts of the immune system to enhance efficacy and durability of the vaccine-mediated response.

### 8.2 Vaccine antigens

Given the vast number of bacterial strains and many diseases caused by UPEC, multiplexing the dominant antigens from the clinically prominent strains and inclusion of an antigen common in all strains of UPEC could be the best approach for effective vaccine against UTI. Since UPEC are ubiquitous in nature and are relatives of commensal bacteria, an effective vaccine strategy that distinguishes commensal and pathogenic *E. coli* is required. Vaccine antigens that target bacterial adhesion factors can impair bacterial colonization; that target bacterial surface molecules can promote bacterial cell recognition and killing, and that target bacterial metabolic proteins will incapacitate bacterial metabolism. However, existence of redundant molecules in bacteria for these processes could undermine the efficacy of vaccine candidates. Nevertheless, a combination of virulence factors in a single vaccine preparation could mitigate this issue.

#### 8.2.1 Polysaccharide antigens

Bacterial polysaccharides on the surface, such as O antigens and the capsule K antigens, are promising vaccine antigens. Conjugation of these antigens to a carrier protein confers enhanced immunogenicity to the polysaccharide antigens, eliciting both T and B cell responses as well as a superior memory response (182–186). Although O and K antigens are major virulence factors involved in *E. coli* pathogenesis, researchers have mostly focused on O antigens. Over 180 distinct O antigens have been described for *E. coli* with seroprevalence of some O antigens (e.g., O25, O2) in specific ExPEC strains isolated from bacteremia patients (187). O antigens are present in a high proportion of clinical isolates (both ExPEC and UPEC) and are amenable for large scale production. Anti-O antibodies are superior in opsonophagocytosis and complement-mediated killing *of E. coli* strains. In addition, K antigens but not O antigens share partial glycolipid sequence homology with human neural tissue (potentially generating tissue cross-reactive antibodies) (183, 188).

#### 8.2.2 Protein antigens

Blocking bacterial cell attachment to host cells by targeting antibodies against fimbria is another strategy to prevent or limit infection. A few clinical studies have demonstrated the potency of FimH vaccines (**Table 2**) as they target a broader spectrum of UPEC strains as all *E. coli* can express FimH (189, 190). FimH is highly conserved molecule among UPEC strains, which makes it a good vaccine candidate. Iron-acquisition proteins are essential for UPEC virulence and metabolic survival. Targeting iron-acquisition proteins and other outer membrane proteins as candidates could impair bacterial metabolic pathways and replication. A combination of O-antigen, adhesion proteins and iron-acquisition proteins as ‘multiple vaccine’ platform could restrain UPEC adhesion, colonization, and replication that will provide adequate protection from UTIs. Whole cell lysates like those used in the Uromune, UroVac, StroVac and Uro-Vaxom vaccines contain a cocktail of multiple antigens by mixing different strains of *E. coli* and other bacteria like Klebsiella. Most of these vaccines have demonstrated a moderate reduction of rUTIs over 12 months (**Table 2**) (17, 18, 165, 166, 191–194). Thus, multiple antigens vaccines might be ideal to provide adequate protection from UTI, through induction of broader neutralizing antibodies engaging at multiple targets of UPEC.

### 8.3 Vaccine adjuvants

#### 8.3.1 Adjuvant mechanisms

Adjuvants act by various mechanisms, such as activating innate immune receptors, recruiting, and activating immune cells, stimulating mucosal cellular immune responses, increasing antigen stability, optimizing antigen delivery, and optimizing pharmacokinetics of the vaccine. The selection of adjuvants has a huge impact on the efficacy of a vaccine. Multiple adjuvants have been studied, and several are approved for use in humans. Historically, adjuvants were designed to trigger innate cells and antigen presenting cells (e.g., DC and macrophages) to inform the adaptive immune cells of antigen presence at the injection site. Appropriate use of an adjuvant is a balancing act because adjuvants could sabotage the innate immune response, leading to either excessive inflammation (reactogenicity) or insufficient priming. The excessive inflammation during vaccination could impair T cell differentiation and memory responses (195). Thus, adjuvant selection should consider the balance between overactivation of the immune system and induction of tolerance. Some adjuvants have been implicated for negative biological effects including autoimmune or autoinflammatory syndrome induced by adjuvants (ASIA or Shoenfeld’s syndrome) (196). Types of commonly used adjuvants and their mechanisms are summarized elsewhere (197).

#### 8.3.2 Pattern recognition receptor and new classes of adjuvants

Although adjuvants can trigger antibody responses in the absence of TLR signaling (198), adjuvants that engage pattern recognition receptors (PRR) such as TLR and NOD like receptors (NLR) could be a good choice to elicit a strong antibody response. Broadly, adjuvants that trigger PRR in many cell types including immune cells and stromal cells could achieve a high activation of T and B cells. New classes of adjuvants with different adjuvant components like bacterial polysaccharides, chemokines, cytokines or nanoparticles have been studied for mucosal vaccines (197). Each component triggers a specific immune response, for example Mincle (Macrophage-inducible C-Type lectin) adjuvant induces a Th1 and Th17 response (199). Adjuvant combinations, in which different adjuvant components are combined, can induce strong and broad immune responses. For example, the cationic adjuvant formulation (CAF)-based liposomes adjuvant CAF09 consists of the c type lectin receptor ligand MMG (synthetic monomycolyl glycerol that binds Mincle) and the TLR ligand Poly-IC produces a Th1, Th17 and strong CD8^+^ T cell response (200). These combination adjuvants can boost specific immune responses and can optimize vaccine efficacy (197).

#### 8.3.3 Adjuvants and antigen interactions

In addition to modulation of local immune response, adjuvants play a critical role in antigen stability, antigen delivery, and pharmacokinetics. Antigen-adjuvant interaction influences stability and immunogenicity. COVID-19 vaccine antigens (RBD subunit) mixed with either Alhydrogel + CpG (a ligand for TLR9) or AdjuPhos + CpG were different in stability where Alhydrogel + CpG was the least stable but induced the strongest antibody response (201). Optimal mixing or the degree of adsorption of vaccine antigens with adjuvants is also critical for the type of immune response elicited. For example, an increased adsorption of antigen to CAF01 adjuvant resulted in higher activation of Th1 and Th17 cells compared with non-absorption of the antigen (202). Therefore, it would be ideal to test multiple adjuvants for their compatibility with the specific antigens to induce the strongest desired mucosal immune response.

### 8.4 Route of administration to induce mucosal immunity

The route of immunization is a critical factor for successful immunization, ideally with minimal reactogenicity for the recipients and easy administration for health care workers. Although direct delivery to the urethral or bladder mucosal sites could be the most effective route for UTI vaccines that could induce local immune response at the urinary tract, it is an unpractical and invasive approach. Intramuscular injection is the most studied route for vaccination against infectious diseases, and the most common route of administration for licensed vaccines. Several other routes of administration have been tried in the clinic with vaccines against UTI (**Table 2**) (165, 184–186, 203).

#### 8.4.1 Sublingual and oral vaccination

Recently, a sublingual vaccination given daily for 3 months has been investigated for UTI (18, 204). The sublingual vaccination route can be an alternate route to intramuscular vaccination since it is easy to administer (needle free) and induces both mucosal and systemic responses with limited adverse effects (205). Further, stable antigen formulations, both in powder and tablet format, can be used for sublingual preparations. The challenge with sublingual application is that antigens can be excessively diluted by saliva and digested by enzymes in the mucosal area and therefore, the amount of antigen required is relatively high (206). Another problem is the level of antigen uptake and processing at sublingual site as underlying oral mucosa lacks organized mucosa associated lymphoid tissue for these processes, and it is considered as a tolerance site due to constant exposure to environmental insult (206). However, the resident CD163^+^ macrophages and some DCs present in the lamina propria of the sublingual mucosa may be sufficient for antigen uptake (207).

#### 8.4.2 Intradermal and subcutaneous vaccination

In addition, intradermal (i.d) or subcutaneous (s.c.) routes are efficient routes for vaccine administration. The i.d. route is shown to be superior compared with i.m. route in inducing mucosal immunity and protection against mucosal diseases such as cystitis, pneumonia, and enteritis, as it activates the expansion of T_FH_ and B cells, but inhibits intermediate monocytes (CD14^+^ CD116^+^) that interfere with classical monocytes (CD14^+^ CD16^-^) (208). As skin has evolved to handle environmental exposure to several microbes, a wide network of APC and effector immune cells in the skin layers could prime a strong vaccine immunity. The i.d. route is shown to be dose- and time-sparing with comparable immune response as that of the i.m. route (209). The s.c. route is another frequently used administration route that renders comparable immune responses to that of i.m. route (210). However, a study comparing reactogenicity of 39 vaccines administered either s.c. or i.m. showed that s.c. route had greater reactogenicity compared with i.m. route (211). Alternatively, a combination of i.m., i.d., and s.c. route of vaccination for prime-boost strategies could be a useful application for effective vaccination against UTI. In humans, concomitant immunization with a DNA vaccine via the i.m. + s.c. routes in a prime-boost setting induced effector T cells (predominantly Th17 cells) with mucosal protection compared with the i.m + i.d. route which induced a Th1 profile (212). Overall, this suggests that different vaccine administration routes are important to consider for mucosal vaccine safety and efficacy.

### 8.5 Correlates of protection for vaccine testing and validation

Correlates of protection (CoP), a positive readout of *protective* immune responses that defend host from invading microbial pathogens, is an indicator or predictor for efficacy of vaccine(s). Although the correlates of protection are not well defined in UPEC infection, it is suggested that the levels of antigen-specific mucosal IgA and serum IgG or tissue kinetics of Th17 and T_RM_ cells could be used as biomarkers of CoP for testing and validation of vaccines (120, 124, 125, 213, 214). A stable and optimal level of mucosal IgA or serum IgG, with effective neutralization, opsonophagocytic or bactericidal activity against UPEC and its adhesion proteins and polysaccharides, is the best predictor of protection. Antibody titers are measured with ELISA, but the functionality measured with an opsonophagocytic killing assay or bactericidal assay is a more critical component to establish a CoP. For example, the CoP for the meningococcal vaccine is the bactericidal assay (215). Serum or urine collected from study participants at pre- and post-vaccination time points, and possibly after natural exposure to UPEC will determine the effective antibody titer in serum and urine that is needed for protection. In the safety and immunogenicity clinical trial for ExPEC4V both antibody titers and functionality of antibodies was measured for all four included O-antigens (186) and this provided valuable information on the immunogenicity of the ExPEC vaccine, however, the CoP for UPEC still needs to be determined. In addition, cellular responses could provide sterilizing immunity at mucosal sites and tissue locality, particularly by destabilizing attachment and biofilm formation. In this regard, Th17 and T_rm_ cells could be key players through production of pro-inflammatory cytokines and constant recruitment of neutrophils to the bladder (123, 125). Therefore, measuring tissue kinetics of Th17 and T_RM_ cells with flow cytometry and effector functions with *in vitro* antigen restimulation in preclinical *in vivo* studies could be essential to identify a preclinical CoP. In addition, measuring IL-17 and other pro-inflammatory cytokines in serum could provide valuable information on cellular immunity upon clinical vaccine trials.

## 9 Discussion

**Figure 4:**
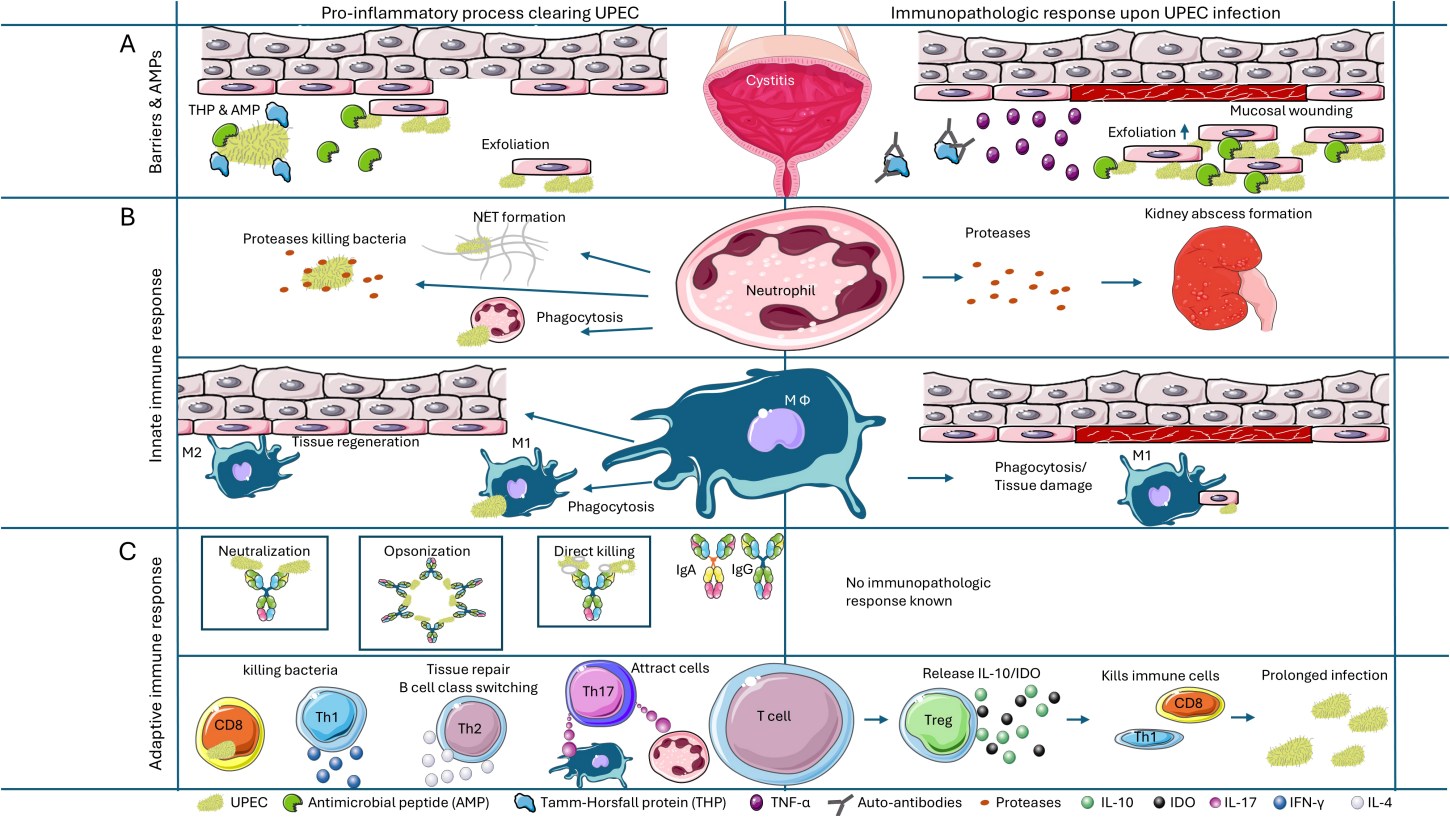
Balancing the pro-inflammatory and immunopathologic response upon *Uropathogenic E. coli* (UPEC) infections. **(A)** Pro-inflammatory side: Exfoliation of infected epithelial cells from the epithelial barrier with antimicrobial peptides and Tamm-Horsfall proteins that bind and block UPEC. Immunopathology side: prolonged overexpression of TNF-α, antimicrobial peptides and auto-antibodies to Tamm-Horsfall proteins induce excessive exfoliation and mucosal wounding. **(B)** Pro-inflammatory side: Innate immune response with neutrophils and macrophages that phagocytose and kill UPEC. Immunopathology side: Proteases from neutrophils induce kidney abscess formation and increase phagocytosis by macrophages leading to tissue damage. **(C)** Pro-inflammatory side: Adaptive immune response with IgG and IgA antibodies inducing neutralization, opsonization and direct killing of UPEC. CD4^+^ and CD8^+^ T cell subsets promote UPEC clearance and tissue repair. Immunopathology side: For UPEC specific antibodies, no immunopathology is described. Regulatory T cells release IDO and IL-10 to dampen the immune response by killing immune cells and this could lead to prolonged infection with more mucosal wounding and edema. Figure created by adapting and using images from Servier Medical Art (https://smart.servier.com/), licensed under CC BY 4.0 (https://creativecommons.org/licenses/by/4.0/).

In this review, we provided a comprehensive summary of the protective versus the pathologic responses activated by UPEC during UTI as captured in **Figure 4** and **Box 1**. We described the first line of defense against UPEC infections with the epithelial barrier, AMPs, and Tamm Horsfall protein. Exfoliation of infected epithelial cells and prevention of binding of UPEC to epithelial cells are the main events at the infection site, while a prolonged overexpression of TNF-α, AMPs and Tamm Horsfall protein can also cause excessive exfoliation resulting in mucosal wounding (as discussed in chapters 3 & 7, **Figure 4A**). The second layer of defense is the innate immune response with neutrophils and macrophages that are the main players to kill bacteria. The neutrophils harness NET formation, proteases, and phagocytosis to kill bacteria, but on the other hand the proteases can also damage the kidneys and cause abscesses. The macrophages can aid in tissue regeneration and phagocytoses of bacteria, but too much phagocytosis of damaged and infected cells leads to tissue damage and mucosal wounding. A delicate balance of these innate players is important to stop the infection and prevent tissue damage (as discussed in chapters 4 & 7, **Figure 4B**). The third line of defense is the adaptive immune system with antibodies and T cells. Antibodies, both IgG and IgA isotypes, initiate different processes upon binding to UPEC bacteria, from neutralization to direct killing. Different subsets of CD4^+^ T cells, including Th1, Th17 and T_RM_ play a protective role, while Th2 promotes urothelium repair and resolves inflammation. On the other hand, mast cells, regulatory T cells and soluble mediators (e.g., IL-10, IDO) negatively impact the immune response by decreasing inflammatory process or silencing immune cell functions and thereby prolong the infection (as discussed in chapters 5 & 7, **Figure 4C**).

Box 1 **Summary of immune response (protective versus immunopathology) and immune pathways activated during *Uropathogenic E. coli* (UPEC) infections**

1. Antimicrobial peptides are beneficial, but cathilicidins can also cause immunopathology by exacerbating exfoliation.
2. Neutrophils are protective, but too many neutrophils can cause immunopathology.
3. Inflammatory macrophages (M1) are phagocytosing UPEC but also cause immunopathology. Alternatively activated macrophages (M2) help in resolution of inflammation and moderation of tissue pathology.
4. Inflammasome (e.g., NLRP3) activation is protective, but inflammasome-independent IL-1β release can cause immunopathology.
5. Antibody response is elicited in pyelonephritis, but the local bladder antibody response is impaired during cystitis.
6. T cells (including Th1, Th17, and T_RM_) are beneficial, but limited information on the role of cytotoxic T cells is available. Th2 cells are shown to impair antibody response and bacterial control but promote tissue repair.

UTI cases are frequently seen in clinics and cause a financial and health care burden. Most of these UTIs are treated with antibiotics. Due to the rise of AMR within the UPEC strains, the success of this treatment is projected to decrease over time. Prevention of UTIs with vaccines seems to be the best path forward. Multiple vaccines, from a single antigen to whole cell vaccines, have been tested in clinical trials but none have been broadly licensed yet. Vaccines with only a single antigen like the FimCH vaccine that targets FimH mediated adherence of *E. coli*, have shown the potential to increase the levels of target-specific functional antibodies in serum, however clinical efficacy of this type of vaccine in phase 3 clinical trials remains to be shown. Multiple *E. coli* antigens might be needed to develop a vaccine that not only inhibits the FimH-mediated adherence of *E. coli* but also activates other immune processes like multiple T helper cell subsets, tissue resident T cells and induces a broad antibody repertoire to prevent infection and colonization. Whole cell vaccines naturally consist of more antigens, and vaccines such as Uro-Vaxom show moderate prophylactic efficacy for 12 months in recurrent UTI patients. Nonetheless, these vaccines induce insufficient immune memory to have a long-lasting effect without the need for additional booster vaccinations. The sublingual whole cell vaccine spray Uromune (MV-140) also showed a reduced recurrence of UTI for 12 months follow-up when used daily for 3 months. However, a broad spectrum in clinical responses, which ranges between 38-90% of patient without recurrent UTIs in 12 months, are seen between vaccine studies with Uromune. Differences in UTI conditions (uncomplicated vs complicated vs recurrent) between studies as well as adherence to the daily dosing could account for the broad spectrum of responses that were seen; however, more research is needed. Another point of discussion is the nature and suitability of antigens that are present on both commensal and pathogenic *E. coli*, such as O antigens, FimH, etc. Only specific O antigens are associated with pathogenic *E. coli* (187) and therefore, the expected impact on the commensal *E. coli* in the microbiome is minimal. For FimH, all *E. coli* can express it if the right environment exists, like in bladder and other mucosal areas, but can also switch to the absence of expression of FimH in other environment (184). However, anti-*E. coli* vaccine(s) might have a potential impact on microbial ecology and human intestinal microbiome. Appreciably, *E. coli* is a highly versatile bacterium, capable of surviving in many environments/habitats, including soil, water, and industrial settings. It is also a minor (0.1%) constituent of the human gut microbiome, where it establishes a symbiotic relationship with the host to help in digestion and take advantage of this habitat for microbiome survival (216). These non-pathogenic commensal *E. coli* strains form a microbial community that is harmless for the gut. Unless pathogenic *E. coli* strains colonize the gut and the urinary tract, commensal strains do not alter healthy mucosal surfaces. This, in part, is due to the regulated immune surveillance that does not attack commensal *E. coli* at the mucosal surfaces. This non-harming immune surveillance may not be silent if the balance is disturbed on symbiotic or commensal relationship, because of use of anti-*E. coli* vaccine for longer term. Although vaccine strategies will be focused on differential antigen expression between commensal and pathogenic *E. coli*, it is possible that vaccine-mediated humoral and cellular responses could have attendant residual effect on commensal bacteria too, after targeted effect on pathogenic *E. coli*. If this residual effect persists, the gut microbiome can be altered, which will eventually alter the microbial community in the ecology. Thus, an interconnected network effect of anti-*E. coli* vaccine on gut microbiome and microbial ecology is possible, in addition to an overarching benefit of limiting the evolution or perpetuation of antimicrobial resistant *E. coli*. Vaccination with FimCH in Cynomolgus monkeys revealed no differences in gut microbiome composition before and after vaccination (217). Currently no data is available on the impact of ExPEC/UPEC vaccination on the human microbiome. Therefore, it is important to add surveillance of the participants microbiome during clinical trials for vaccines against UPEC.

It is suggested that for effective control and clinical management of UTI/rUTI, anti-UPEC vaccine is the best alternative next to antibiotics. This approach could also prevent the emergence of anti-microbial resistant bacteria. However, a right combination of vaccine antigens and adjuvants is needed for a strong immune response. Adjuvants with a multitude of effects from stimulating different subsets of T helper cells and B cells to boost antibodies at mucosal surface is important step in developing vaccine against UPEC. Newer adjuvants have recently become available that combine multiple components. However, the safety and efficacy profile of these adjuvants needs validation in preclinical and clinical models. Initiatives to make adjuvants available more broadly for research and development communities are underway and will stimulate the introduction of these newer adjuvants in the clinical space. Antigen stability and presentation of the antigens in combination with the adjuvants remain to be studied. Immune response can be different for a single antigen when combined with different adjuvants. Thus, for an effective vaccine, it is advisable to test multiple adjuvants for their compatibility with the specific antigens and their ability to induce a mucosal immune response in the bladder.

A body of work in the past several decades on UTIs and UTI vaccine development has demonstrated the critical roles of innate, cellular and antibody responses to contain UTIs. Despite this gained knowledge, limited progress has been made to advance UPEC vaccine(s) for prevention of UTI. This is due to the complexity of the host-bacterial interaction together with the unknown correlate(s) of protection in UTI. As discussed above, *E. coli* strains colonize vertebrate gut and other mucosal surfaces, while eliciting an array of responses upon host-pathogen interaction. Notably, *E. coli* exploits host system for successful colonization and pathogenesis, which involve multiple and complex mechanisms that are impenetrable by manual intervention strategies. In addition, the variable and transient host responses elicited by *E. coli* makes it harder to focus on a consistent CoP during vaccine development across different intervention platforms. Importantly, *E. coli* is adept in survival with limited metabolic activity on non-host surfaces such as soil and water, enabling differential gene expressions that make it highly adaptable to adverse environments including antibiotic-rich or host-defense bactericidal machineries. However, a successful development of an effective UPEC vaccine will prevent occurrence or recurrence of UTI. Further, a regular clinical adoption of UPEC vaccine will also limit the development and progression of anti-microbial resistant bacteria. This will bring a long-term positive impact to the healthcare sectors worldwide, and benefit the most vulnerable populations (e.g., children, elderly adults, and UTI-susceptible individuals).
